# Evaluation of body fat content and osteoarthritis in cats using computed tomography – a novel approach using whole-body imaging

**DOI:** 10.1186/1751-0147-57-S1-P7

**Published:** 2015-09-25

**Authors:** Cecilia Ley, Charles J Ley

**Affiliations:** 1 Department of Biomedical Sciences and Veterinary Public Health, Swedish University of Agricultural Sciences, Uppsala, Sweden; 2 Department of Clinical Sciences, Swedish University of Agricultural Sciences, Uppsala, Sweden

## Introduction

Development of whole-body multidetector computed tomography (MDCT) methods have the potential to allow investigations into relations between feline osteoarthritis (OA) and obesity. In one MDCT examination all joints in an animal and the total body fat content can be evaluated. However, studies investigating the correlation between presence of joint lesions detected by MDCT and macroscopic evidence of OA are lacking.

## Objectives

The aims of the study were to evaluate whole-body MDCT for the diagnosis of feline OA, and to utilize a method for whole-body MDCT-based quantitative fat content determination.

## Methods

MDCT images from 30 cats were evaluated for joint lesions and fat percentages (fat%) were calculated for 14 cats. Body condition scores (BCS) were determined using a nine-grade scale. The shoulder, elbow, antebrachiocarpal, hip, stifle and tarsocrural joints were macroscopically evaluated for presence of cartilage lesions. Associations between MDCT-detected lesions and macroscopic cartilage lesions were investigated, and the correlation between MDCT-fat% and BCS determined.

## Results

Significant associations between MDCT-detected lesions and macroscopic cartilage lesions were detected in the shoulder (p=0.0002), elbow (p=0.009), and tarsocrural (p=0.004) joints. MDCT estimates of fat% ranged from 13.4-48.6% (median 34%) and the BCS ranged from 2-8 (median 5). There was significant correlation between fat% and the BCS (p=0.006).

## Conclusion

Whole-body MDCT is useful for predicting macroscopic cartilage lesions in feline shoulder, elbow and tarsocrural joints and for determination of body fat content. These methods may be valuable for future studies of feline OA and investigations into the possible influence of obesity on OA development.

